# A Nationwide Study about the Dispersal Patterns of the Predominant HIV-1 Subtypes A1 and B in Greece: Inference of the Molecular Transmission Clusters

**DOI:** 10.3390/v12101183

**Published:** 2020-10-19

**Authors:** Evangelia Georgia Kostaki, Maria Gova, Georgios Adamis, Georgios Xylomenos, Maria Chini, Nikos Mangafas, Marios Lazanas, Simeon Metallidis, Olga Tsachouridou, Vasileios Papastamopoulos, Dimitrios Chatzidimitriou, Eleni Kakalou, Anastasia Antoniadou, Antonios Papadopoulos, Mina Psichogiou, Dimitrios Basoulis, Dimitrios Pilalas, Ifigeneia Papageorgiou, Dimitra Paraskeva, Georgios Chrysos, Vasileios Paparizos, Sofia Kourkounti, Helen Sambatakou, Vasileios Bolanos, Nikolaos V. Sipsas, Malvina Lada, Emmanouil Barbounakis, Evrikleia Kantzilaki, Periklis Panagopoulos, Vasilis Petrakis, Stelios Drimis, Charalambos Gogos, Angelos Hatzakis, Apostolos Beloukas, Lemonia Skoura, Dimitrios Paraskevis

**Affiliations:** 1 Department of Hygiene, Epidemiology and Medical Statistics, Medical School, National and Kapodistrian University of Athens, 11527 Athens, Greece; ekostakh@med.uoa.gr (E.G.K.); marygova@gmail.com (M.G.); ifipap88@gmail.com (I.P.); ahatzak@med.uoa.gr (A.H.); 2 1st Department of Internal Medicine, G. Gennimatas General Hospital, 11527 Athens, Greece; geo.adamis@gmail.com (G.A.); g.xylomenos@gmail.com (G.X.); 3 3rd Department of Internal Medicine-Infectious Diseases Unit, “Korgialeneio-Benakeio” Red Cross General Hospital, 11526 Athens, Greece; mariachini@gmail.com (M.C.); nikosmangafas@gmail.com (N.M.); mklazanas@ath.forthnet.gr (M.L.); 4 1st Department of Internal Medicine, AHEPA University Hospital, Medical School, Aristotle University of Thessaloniki, 54636 Thessaloniki, Greece; metallidissimeon@yahoo.gr (S.M.); olgat_med@hotmail.com (O.T.); 5 5th Department of Internal Medicine and Infectious Diseases, Evaggelismos General Hospital, 10676 Athens, Greece; vasileios.papastamopoulos@gmail.com (V.P.); ekakalou@yahoo.gr (E.K.); 6 National AIDS Reference Centre of Northern Greece, Department of Microbiology, Medical School, Aristotle University of Thessaloniki, 54124 Thessaloniki, Greece; dihi@med.auth.gr (D.C.); pilalas_jim@hotmail.com (D.P.); mollyskoura@gmail.com (L.S.); 7 4th Department of Medicine, Attikon General Hospital, Medical School, National and Kapodistrian University of Athens, 12462 Athens, Greece; ananto@med.uoa.gr (A.A.); antpapa1@otenet.gr (A.P.); 8 1st Department of Medicine, Laikon General Hospital, Medical School, National and Kapodistrian University of Athens, 11527 Athens, Greece; mpsichog@yahoo.gr (M.P.); dimitris.bassoulis@gmail.com (D.B.); 9 Department of Internal Medicine, Tzaneio General Hospital, 18536 Piraeus, Greece; dimparaskeva@gmail.com (D.P.); gchrysos@gmail.com (G.C.); steliosdrimis@gmail.com (S.D.); 10 HIV/AIDS Unit, A. Syngros Hospital of Dermatology and Venereology, 16121 Athens, Greece; vpaparizos@yahoo.gr (V.P.); kourkounti@in.gr (S.K.); 11 HIV Unit, 2nd Department of Internal Medicine, Hippokration General Hospital, Medical School, National and Kapodistrian University of Athens, 11527 Athens, Greece; helensambatakou@msn.com (H.S.); billy_bolanos@hotmail.com (V.B.); 12 Department of Pathophysiology, Laikon General Hospital, Medical School, National and Kapodistrian University of Athens, 11527 Athens, Greece; nsipsas@med.uoa.gr; 13 2nd Department of Internal Medicine, Sismanogleion General Hospital, 15126 Marousi, Greece; malvinalada@gmail.com; 14 Department of Internal Medicine, University Hospital of Heraklion “PAGNI”, Medical School, University of Crete, 71110 Heraklion, Greece; barbuman2003@yahoo.gr (E.B.); eyrykleia3@yahoo.gr (E.K.); 15 Department of Internal Medicine, University General Hospital, Democritus University of Thrace, 68100 Alexandroupolis, Greece; ppanago@med.duth.gr (P.P.); vasilispetrakis1994@gmail.com (V.P.); 16 Department of Internal Medicine and Infectious Diseases, University Hospital of Patras, 26504 Rio, Greece; cgogos@med.upatras.gr; 17 Department of Clinical Infection, Microbiology and Immunology, Institute of Infection and Global Health, University of Liverpool, Liverpool L697BE, UK; 18 Department of Biomedical Sciences, School of Health Sciences, University of West Attica, 12243 Athens, Greece

**Keywords:** human immunodeficiency virus (HIV), molecular epidemiology, phylogenetic analysis, phylogeographic analysis, local transmission, dispersal patterns, transmission clusters, Greece

## Abstract

Our aim was to investigate the dispersal patterns and parameters associated with local molecular transmission clusters (MTCs) of subtypes A1 and B in Greece (predominant HIV-1 subtypes). The analysis focused on 1751 (28.4%) and 2575 (41.8%) sequences of subtype A1 and B, respectively. Identification of MTCs was based on phylogenetic analysis. The analyses identified 38 MTCs including 2–1518 subtype A1 sequences and 168 MTCs in the range of 2–218 subtype B sequences. The proportion of sequences within MTCs was 93.8% (1642/1751) and 77.0% (1982/2575) for subtype A1 and B, respectively. Transmissions within MTCs for subtype A1 were associated with risk group (Men having Sex with Men vs. heterosexuals, OR = 5.34, *p* < 0.001) and Greek origin (Greek vs. non-Greek origin, OR = 6.05, *p* < 0.001) and for subtype B, they were associated with Greek origin (Greek vs. non-Greek origin, OR = 1.57, *p* = 0.019), younger age (OR = 0.96, *p* < 0.001), and more recent sampling (time period: 2011–2015 vs. 1999–2005, OR = 3.83, *p* < 0.001). Our findings about the patterns of across and within country dispersal as well as the parameters associated with transmission within MTCs provide a framework for the application of the study of molecular clusters for HIV prevention.

## 1. Introduction

HIV remains a major health challenge to date. Although HIV transmissions have declined over the last few years, incidence remains high (http://www.UNAIDS.org). According to the UNAIDS/WHO, 1.7 million people became newly infected with HIV in 2019 (http://www.UNAIDS.org). HIV-1 genetic diversity is a hallmark of the virus and since the beginning of the epidemic, group M of HIV-1 has been genetically classified into pure subtypes (A–D, F–H, J–L), sub-subtypes, recombinant forms, and unclassified viruses [1,2]. The recombinant forms of the virus are further divided into circulating recombinant forms (CRFs) and unique recombinant forms (URFs) [1]. The prevalence of HIV-1 subtypes and recombinants varies greatly with the subtype C being predominant, followed by subtypes B, A, CRF02_AG, and CRF01_AE [3]. In the absence of an effective vaccine and cure, prevention of HIV-1 transmission provides the only strategy to mitigate its impact and reduce incidence (http://www.UNAIDS.org).

Phylogenetic analyses of HIV-1 have several applications in public health, including investigation of transmission dynamics and dispersal and outbreak investigation [4,5,6,7,8,9,10,11,12,13,14,15,16]. Recently, the Centers for Disease Control and Prevention (CDC) has discussed the benefits of HIV cluster analysis for public health action [17]. Investigation on HIV molecular clusters can provide important data about the dispersal patterns of the epidemic, as well as the characteristics of people living with HIV (PLHIV) infected within clusters [4,5,6,7,8,9,11,12,13,14,15,16,18]. Moreover, by investigating the molecular clusters, we can identify those at higher risk for HIV infection (e.g., the social links of recently infected PLHIV through contact tracing programs) and intervene with targeted strategies to prevent HIV [17].

HIV cluster analysis has been performed in many instances and there is increasing evidence that this knowledge can be useful for public health purposes. The benefits of cluster analysis include the ability to identify clustered infections that occurred recently and, therefore, focus on populations at higher risk of getting or transmitting HIV [17]. This knowledge can be extracted from data generated for HIV drug resistance testing, which is being performed widely.

Our previous studies have shown that subtypes A1 and B are the most prevalent, with the former following an increasing trend after 2000 in Greece [19]. The outbreak among people who inject drugs (PWID) in the Athens metropolitan area that ignited in 2011 was a different situation, where most viruses were classified as CRF14_BG followed by CRF35_AD and subtypes A1 and B [15,20]. The local clusters were of considerable importance for the high prevalence of non-nucleoside reverse transcriptase inhibitors (NNRTI) resistance mutations in Greece, where the majority of the most prevalent resistance mutations (e.g., E138A/G, K103N, Y181C) were due to onward transmissions within molecular transmission clusters (MTCs) [21].

Our aim was to provide a framework of HIV cluster analysis for public health action in Greece, by means of molecular epidemiology. Specifically, we aimed to identify the MTCs of subtypes A1 and B, which are the most prevalent clades in Greece, and to investigate the parameters associated with infections within MTCs. We also aimed to infer the within country patterns of HIV-1 dispersal.

## 2. Materials and Methods

### 2.1. Study Population

The study dataset included 6166 HIV-1 sequences available in the protease and partial reverse transcriptase regions (*pol* gene) obtained from PLHIV during 1999–2015 in Greece. This number accounts for 57.2% of all people diagnosed with HIV-1 during the same period in Greece (*n* = 10,787). Surveillance data were available from the National Public Health Organization (formerly, Hellenic Centre for Disease Control and Prevention) [22]. Specifically, our study sample consisted of 4790 (77.7%) sequences generated in Athens (sampled from Central/Southern Greece) and 1376 (22.3%) sequences generated in Thessaloniki (1298 (94.3%) sampled from Northern Greece and 78 (5.7%) from Crete). In the case of multiple available sequences per patient (sequences collected at different time points during the patient’s follow-up), only the earliest available sequence was included in the analysis. A unique serial number was used to specify each patient, while duplicate patients were detected and excluded from the study population. The current project has been approved by the Ethics Committee of the Medical School, National and Kapodistrian University of Athens.

### 2.2. Subtyping Analysis

HIV-1 subtyping was carried out by using the online automated subtyping tools COMET (COntext-based Modeling for Expeditious Typing) (http://comet.retrovirology.lu) and REGA (http://dbpartners.stanford.edu/RegaSubtyping/html/indexhbv.html). In addition, the study sequences (*n* = 6166) were analyzed phylogenetically along with a set of 216 reference sequences representative of all pure subtypes, sub-subtypes, and the majority of CRFs, collected from the HIV-1 sequence database (http://www.hiv.lanl.gov/). Sequence alignment and editing were performed on MEGA X [23]. Editing was conducted manually and according to the coding reading frame. Phylogenetic analysis was performed by the approximate maximum likelihood (ML) method, as implemented in FastTree v2.1 [24], using Generalized Time Reversible (GTR) as a substitution model with CAT approximation. Tree visualization and annotation were performed on the programs Dendroscope v3.5.7 (http://dendroscope.org) and FigTree v1.4 (http://tree.bio.ed.ac.uk/software/figtree/). Furthermore, all the study sequences which remained unclassified from the previous procedures were tested for the presence of recombination by using the bootscanning approach as implemented in SimPlot v3.5.1 [25]. HIV-1 subtyping confirmation by phylogenetic and recombination analysis has focused on the characterization of subtypes A1 and B, which are the most prevalent clades in Greece, as described previously [19,21].

### 2.3. Phylogenetic Analysis

The dispersal patterns of HIV-1 subtypes A1 and B in Greece were investigated by means of phylogenetic analysis. Analysis was performed separately for each subtype and was repeated in five replicates using a different set of randomly selected references, in order to minimize the possibility of overrepresentation of countries or geographic regions with a higher number of available sequences due to more frequent HIV drug resistance testing. Specifically, sequences from our study population (A1: 1751; B: 2575) were analyzed along with a randomly selected globally sampled dataset of sequences (A1: 1500; B: 2000), used as references. Reference sequences were available in the HIV-1 sequence database and their subtype was checked and confirmed by COMET. The phylogenetic trees were inferred by the ML method under the GTR + G4 substitution model as implemented in RAxML v8.2.10 [26].

Thereafter, phylogenetic clusters consisting of at least two sequences sampled from Greece at a proportion greater than 70% compared to the total number of sequences within the cluster (geographic criterion) were defined as tentative MTCs [27,28]. Tentative MTCs that were present and fulfilled the geographic criterion in all five rounds of phylogenetic analysis were defined as MTCs. Robustness of all the large MTCs (i.e., MTCs including more than 10 sequences) was confirmed by ML transfer bootstrap observation (TBE) and Bayesian phylogenetic analysis [29]. Specifically, phylogenetic analysis using either ML or Bayesian methods were performed for each individual MTC using as references the most closely related sequences identified after a BLAST search using the HIV BLAST tool (http://www.hiv.lanl.gov). Only unique sequences identified using 10 BLAST matches to each query were included in the analysis. ML phylogenetic analysis was conducted by the RAxML v8.2.10 program, as specified in the above paragraph. Bayesian analysis was performed by using MrBayes [30] with GTR + G and run for 10 × 10^6^ generations (burnin: 10%). The convergence of all parameters of the Markov chain Monte Carlo (MCMC) was assessed by Tracer [31], if the effective sample size (ESS) was higher than 200. MTCs receiving bootstrap support > 75% or posterior probability support > 0.85 were considered as significant.

### 2.4. Phylogeographic Analysis

In order to infer the within country patterns of HIV-1 dispersal, we performed phylogeographic analysis on all the large MTCs of subtypes A1 and B. Specifically, phylogeographic analysis was performed separately on the MTCs of each subtype over a high number of bootstrap trees (*n* = 408), reconstructed by ML phylogenetic analysis that was conducted on the RAxML v8.2.10 program. Initially, we tested if the HIV transmissions (viral migration events) occurred more frequently among PLHIV from the same geographic area (Central/Southern Greece, Northern Greece, Crete) in a significant manner. The hypothesis testing was performed by character reconstruction using the criterion of parsimony on Mesquite v3.61 [32]. Afterwards, we quantified the viral migration events between the different geographic areas by using PAUP*4.0 [33], and we assessed if they were higher than those expected by chance (panmixis). Further details about the way of estimating viral migration events has been described in detail elsewhere [34,35,36].

### 2.5. Statistical Analysis

Mean and standard deviation (SD)/median and interquartile range (IQR) for continuous variables and absolute and relative frequencies for categorical variables were used to summarize the demographic data of the study. The non-parametric Mann–Whitney U test was used for the comparisons of the relevant distributions, by defining the level of significance at 5% and by adjusting the level of significance according to the Bonferroni correction for multiple comparisons. The demographic parameters associated with the local transmission of subtypes A1 and B in Greece were estimated by multivariable logistic regression models that were fit to a subset of the original data consisting of complete observations (A: 1635; B: 2453). Presence in MTCs was the binary outcome variable, while age, gender, transmission risk group, origin, sampling period, and area of sampling were chosen as possible explanatory variables in both models. Statistical analyses were performed on STATA 13.1 (StataCorp LP).

### 2.6. Sequences Availability

The whole dataset corresponds to a dense sampling of PLHIV in Greece. Therefore, to avoid the risk of PLHIV identification, sequences will be available upon reasonable request.

## 3. Results

### 3.1. Subtyping Results

Subtyping analysis performed by COMET and REGA on our study sequences (*n* = 6166) revealed that the vast majority of the sequences were classified into subtypes B (2575; 41.8%) and A1 (1751; 28.4%). CRF14_BG (469; 7.6%), subtype C (175; 2.8%), CRF35_AD (160; 2.6%), CRF56_cpx (148; 2.4%), and CRF02_AG (144; 2.3%) were also detected at low prevalence. In addition, 153 (2.5%) sequences were found to belong to other subtypes and 173 (2.8%) sequences to other CRFs, while the rest of them (418; 6.8%) were classified as recombinant or unclassified forms of the virus.

Our analysis has focused on the detection of the two most prevalent clades (A1 and B) in Greece. The demographic characteristics of the PLHIV infected with subtypes A1 and B are presented in detail in Table 1. In both subtypes, most of the PLHIV were males (B: 86.1%; A1: 81.9%) of Greek origin (B: 82.2%; A1: 75.4%) with similar age (in years, median values: B: 36; A1: 37). In addition, the predominant mode of infection in this population was through sex between men (Men having Sex with Men—MSM) (B: 66.7%; A: 59.1%). A higher proportion of heterosexuals were found among those infected with subtype A1 (22.1%) versus subtype B (17.0%). Furthermore, a significant decreasing trend was observed for the prevalence of subtype B over the study period (1999–2015) (*p* = 0.001), using the sampling years of the sequences as a proxy of the diagnosis years of the treatment-naive PLHIV infected with subtype B. The decreasing trend of subtype B was also found to be significant over the time period between 2005 and 2015, when HIV drug resistance testing was performed routinely on treatment-naïve PLHIV and the sampling coverage was higher (*p* = 0.012). No significant trend was observed over time for subtype A1 (1999–2015: *p* = 0.410; 2005–2015: *p* = 0.139).

### 3.2. Subtypes A1 and B Dispersal Patterns and Regional Transmission

Phylogenetic analysis using large sets of randomly selected reference sequences was performed in five replicates for each one of the most prevalent subtypes (A1 and B) in order to characterize the spread of the HIV-1 epidemic in Greece. Further analysis confirmed the phylogenetic credibility of MTCs with more than 10 sequences (large MTCs). A new version of phylogenetic bootstrap (TBE) was selected since with large phylogenies, the classical Felsenstein’s bootstrap proportion (FBP) tends to give low support, especially at deep branches [29]. Estimation of MTCs revealed the levels of regional transmission per subtype by dividing the number of HIV-1 sequences from Greece found within MTCs with the total number of available sequences from Greece. Levels of regional transmission corresponds to PLHIV who have been infected locally.

**Figure 1:**
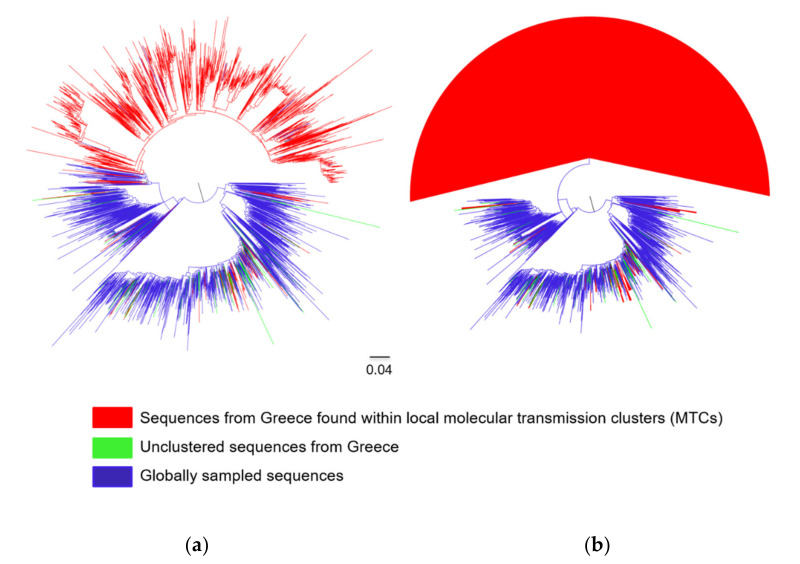
Unrooted phylogenetic trees estimated by RAxML v8.2.10 of HIV-1 subtype A1 sequences from Greece and a global reference dataset. (**a**) Sequences from Greece are marked in light green (unclustered sequences) and red (sequences found within local molecular transmission clusters-MTCs) in contrast with sequences from other countries marked in blue. (**b**) The MTCs are indicated as triangles.

**Figure 2:**
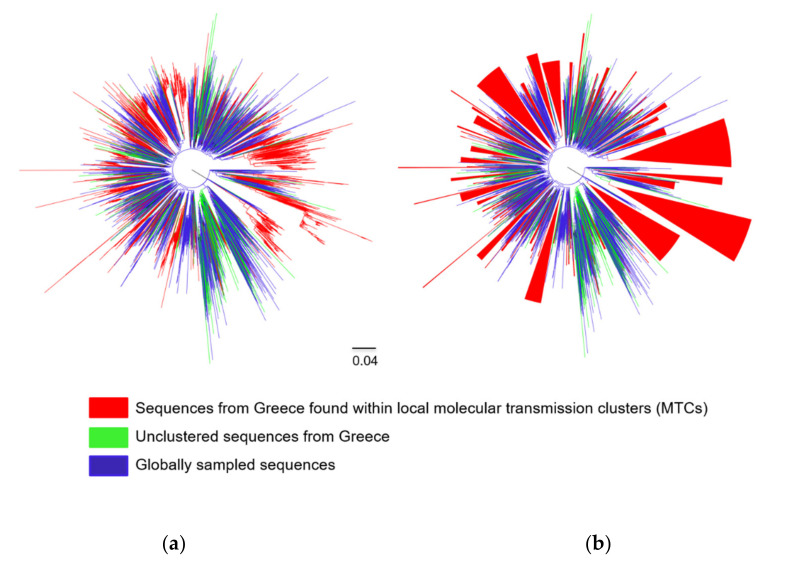
Unrooted phylogenetic trees estimated by RAxML v8.2.10 of HIV-1 subtype B sequences from Greece and a global reference dataset. (**a**) Sequences from Greece are marked in light green (unclustered sequences) and red (sequences found within local molecular transmission clusters-MTCs) in contrast with sequences from other countries marked in blue. (**b**) The MTCs are indicated as triangles.

Analyses revealed that the subtype A1 and B sequences from Greece clustered at different points in the ML trees (Figure 1a and Figure 2a). In addition, it was found that a considerable number of clusters fulfilled the criteria to be considered as MTCs for both subtypes. Specifically, 93.8% (1642 of 1751) of subtype A1 sequences formed 38 MTCs, with a range of 2–1518 sequences (Figure 1b and Figure S1a). For subtype A1, a large MTC was identified (Figure 1b), while for subtype B, several smaller MTCs were detected across the tree (Figure 2b and Figure S1b). Even though the median number of subtype A1 sequences per MTC was 2 (IQR: 2–4), 86.7% (1518) of the subtype A1 sequences fell within a large single MTC (Figure 1b). This finding indicates that the subtype A1 epidemic in Greece is highly monophyletic and corresponds to transmissions that occurred locally. This large single MTC received high TBE bootstrap support (99%), and according to the sensitivity analysis that was performed previously [19], it is a single outbreak. The evolutionary distances in this MTC were quite large since this strain has been introduced at the early stage of the HIV-1 epidemic in Greece. Specifically, the date of the most recent common ancestor of the subtype A1 in Greece was estimated to be 1977.9 (95% highest posterior density interval: 1973.7–1981.9) [19]. In addition, most of the PLHIV infected within the large single MTC of subtype A1 were males (*n* = 1305; 86.0%) of Greek origin (*n* = 1219; 80.3%) with a median age of 36 years (IQR: 29–46). The predominant mode of infection in this population was through sex between men (MSM) (*n* = 999; 65.8%), while most of the sequences from these PLHIV were sampled from Central/Southern Greece (*n*= 1066; 70.2%). Furthermore, phylogenetic analysis of subtype B sequences revealed the existence of 168 MTCs with a range of 2–218 sequences (Figure 2b and Figure S1b). The proportion of subtype B sequences within MTCs was 77.0% (1982 of 2575). The high levels of dispersal of subtype B (Figure 2a) and the existence of a large number of MTCs (Figure 2b) indicate that the introduction of this subtype into Greece has occurred from multiple sources.

The number of subtype A1 and B introductions into Greece was calculated separately for each subtype by adding the number of sequences found outside the MTCs (unclustered sequences—each corresponding to an independent introduction) and the number of MTCs (e.g., for two MTCs including 5 and 10 sequences, we counted only two introductions—as the number of founder strains within each MTC). The number of introductions was found equal to 147 (38 MTCs + 109 unclustered sequences) and 761 (168 MTCs + 593 unclustered sequences) for subtypes A1 and B, respectively. The proportion of founder strains transmitted locally (MTCs) was similar for subtype A1 (25.8%; 38/147) and B (22.1%; 168/759).

### 3.3. Characteristics of Transmissions Within and Outside MTCs

Our analysis revealed that PLHIV whose sequences found within the MTCs (infected locally) were mostly male (A1: 1373; 83.6%/B: 1729; 87.2%), MSM (A1: 1022; 62.2%/B: 1373; 69.3%), and of Greek origin (A1: 1283; 78.1%/B: 1635; 82.5%) (percentages correspond to PLHIV infected within MTCs) (Table 2). For both subtypes, PLHIV with a more recent diagnosis were more frequently found within MTCs (A1: 2011–2015: 50.5%; 2006–2010: 28.0%; 1999–2005: 21.3%/B: 2011–2015: 41.5%; 2006–2010: 25.7%; 1999–2005: 32.6%) (Table 2).

PLHIV infected with subtype A1 outside MTCs (*n* = 109) were reported mostly as heterosexuals (48; 44.0%), PWID (26; 23.9%), of non-Greek origin (47; 43.1%), and with an available sequence with a more recent sampling time (2011–2015: 45.9%; 2006–2010: 40.4%; 1999–2005: 13.7%) (percentages were estimated for PLHIV infected outside MTCs) (Table 2). For subtype B, infections outside MTCs were more frequent for males (488; 82.3%), MSM (345; 58.2%), PLHIV of Greek origin (482; 81.3%), and PLHIV with an available sequence with an earlier sampling time (2011–2015: 18.9%; 2006–2010: 18.2%; 1999–2005: 62.9%).

### 3.4. Factors Associated with Local Transmission of Subtypes A1 and B

The results of the statistical analysis (multivariable logistic regression models) for the identification of demographic factors associated with local transmission of subtypes A1 and B in Greece are presented in detail in Table 3. Analysis revealed that transmissions within MTCs of subtype A1 were associated with the MSM risk group (MSM vs. heterosexuals, OR = 5.34, *p* < 0.001) and Greek origin (Greek vs. non-Greek origin, OR = 6.05, *p* < 0.001), having been adjusted for the rest of the incorporated variables (Table 3). For subtype B, local transmissions were associated with Greek origin (Greek vs. non-Greek origin, OR = 1.57 *p* = 0.019) and more recent sampling (sampling period: 2011–2015 vs. 1999–2005, OR = 3.83, *p* < 0.001) (Table 3). In addition, it was found that younger age was also associated with an increased probability of local transmission of subtype B (OR = 0.96, *p* < 0.001) (Table 3).

### 3.5. Patterns of Geographical Dispersal

**Figure 3:**
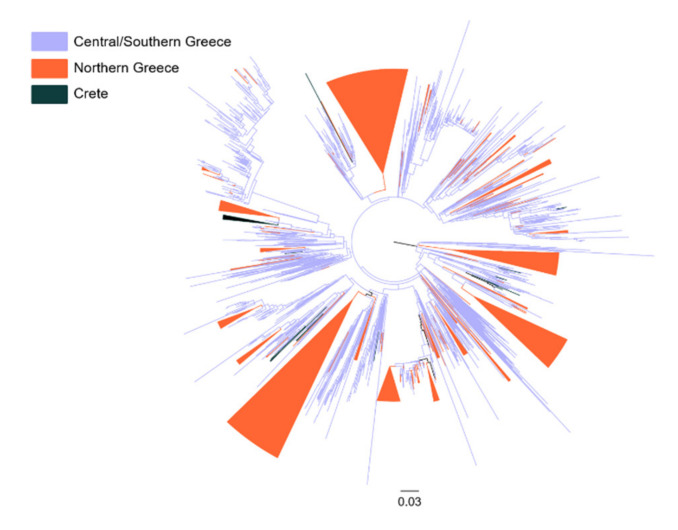
Unrooted phylogenetic tree estimated by RAxML v8.2.10 of HIV-1 subtype A1 sequences from Greece that fell within the large single molecular transmission cluster (MTC). Sequences from Central/Southern Greece are marked in light blue, sequences from Northern Greece in orange, and sequences from Crete in dark green. Clusters consisting of sequences from Northern Greece and Crete are indicated as triangles.

To investigate the patterns of virus spread among different geographic areas in Greece, we performed phylogeographic analysis on the sequences found within MTCs that were generated in Athens and sampled from most areas in Greece (area 1), were generated in Thessaloniki and sampled from Northern Greece (area 2), and a small sample of sequences from Crete also generated in Thessaloniki (area 3). Phylogeographic analyses suggested that the total number of viral migration events corresponding to transmissions among the three different areas was 181 (median value; IQR: 178–184) and 136 (median value; IQR: 134–138) for subtypes A1 and B, respectively. These numbers were significantly lower than the total number of viral migrations expected by chance under the simulated scenario of panmixis (i.e., random mixing of sequences assuming that the level of regional transmission is negligible). Specifically, the total number of viral migrations under panmixis were 398 (median value; IQR: 394–402) and 259 (median value; IQR: 257–262) for subtypes A1 and B, respectively, and were significantly higher than the estimated values for the bootstrap trees for both subtypes (*p* < 0.001). These findings suggest that transmissions occur at the regional level; a conclusion that can also be drawn by the visual inspection of the trees, where the existence of several regional clusters is obvious (Figure 3).

Additionally, we quantified the viral migration events among the different areas and we assessed if they were higher than those expected by chance under panmixis. According to the migration matrix (Table 4), the number of transmissions that were exported from Central/Southern Greece to the other localities (Northern Greece: subtype A1—90.9; subtype B—67.6/Crete: subtype A1—11.6; subtype B—15.0) was lower than those expected by chance, and only transmissions exported from Northern Greece to Central/Southern Greece (subtype A1: 35.4; subtype B: 17.4) were significantly higher than panmixis. Similar results were found between Crete and Central/Southern Greece; however, the number of transmissions was very low (subtype A1: 0.1; subtype B: 1.2) (Table 4).

## 4. Discussion

In the current study, we investigated the patterns of HIV-1 transmission across and among different geographic areas, and we estimated the parameters associated with regional transmission for the two most prevalent subtypes in Greece. We found that a high proportion of subtypes A1 and B, which are the predominant clades, occur locally at 93.8% and 77.0%, respectively. These provide some of the largest proportions of regional transmissions compared to approximately 50% that was reported previously [18,37,38,39,40]. Possible explanations are the dense sample used in our analysis (our study population represents the 57.2% of the total population of PLHIV diagnosed in Greece during 1999–2015) and the method implemented for the identification of MTCs that was based on phylogenetic analysis using a novel bootstrap approach, which is more appropriate for large datasets [29]. Moreover, we did not implement a genetic threshold as a criterion for the identification of MTCs, since our aim was to identify not only PLHIV with close transmission links, but all individuals infected locally. For subtype B, where the dispersal pattern was not monophyletic as was found for subtype A1, the regional transmissions were estimated equal to 77.0%, which is much higher than the estimations of previous studies (reasons mentioned above). Notably, Greece provides one of the few countries in Europe where subtype A1 is found at high prevalence and is associated with the local population and not with the PWID risk group [19,41]. Similarly, it has been reported for Albania and Cyprus [39,42], while for most countries in Western and Central Europe, subtype C and recombinant forms are the most prevailing viruses [3]. The subtype A1 epidemic in Greece differs from the A6 epidemic in Russia and Eastern European countries, where the majority of transmission have been found among the PWID and the origin of infection is from a distinct route [19,43,44,45,46,47]. Furthermore, a recent study showed that Greece provides the main source of subtype A1 transmissions in Europe and remote locations, such as the USA and Australia [48].

The proportion of subtype A1 and B introductions leading to MTCs were similar, suggesting that approximately one-quarter of cases were associated with different levels of onward transmissions in MTCs. These findings indicate that one of the reasons why the non-B subtypes are less frequently associated with transmission chains is probably that they are less abundant in European countries than subtype B [49] (http://www.hiv.lanl.gov). Although the estimated proportion of strains leading to MTCs can be extrapolated as a proportion of onward transmission for the whole population infected within the MTCs, it provides a proxy of the proportion of cases providing as sources for HIV infection. Given that our study population is a subsample of the total number of PLHIV in Greece, the previous proportion should be considered as the minimum estimate.

Our analysis suggested that regional transmission was associated for both subtypes with Greek origin, while the MSM risk group was identified as an additional associated parameter only for subtype A1 and more recent diagnosis and younger age for subtype B, respectively. These findings are in accordance with findings from Europe and the USA, where the MSM risk group, male gender, younger age, and recent diagnosis were the main factors associated with HIV-1 infections within MTCs [18,37,38,50,51,52], suggesting that prevention efforts should focus on populations with these characteristics. The exact effect of the time of sampling to MTCs cannot be estimated, since for sequences sampled earlier, identifying their transmission links is less likely and therefore, the likelihood is lower to be placed in MTCs [18].

Regarding the dispersal patterns of the virus, our analyses focused on sequences found within MTCs and we found that regional dispersal dominated within the geographic areas of study. This finding was supported by the significantly lower number of migration events found among the different areas as compared to the migration events expected under the scenario of random mixing of the sequences. The quantification of viral migration events among the different localities suggested a limited number of transmissions from the largest area to the two others, whereas transmissions from Northern Greece to Central/Southern Greece were higher than expected by chance. We should note that the number of transmissions from Central/Southern Greece to the other areas was the largest, but according to statistical phylogeography, they were less than those expected under the random mixing scenario. The high numbers of transmissions from Central/Southern Greece were probably due to the larger sample size of this area compared to Northern Greece or Crete; however, these numbers were lower than what was expected by chance. These findings indicate that Northern Greece, although it is smaller in sample size than Central/Southern Greece, transmits the virus at high rates to the latter area. Therefore, besides the major local dispersal pattern observed within the three different areas, some cross-regional transmissions occur, with Northern Greece acting as a major source for this type of viral dispersal. A similar pattern has been observed for the dispersal of the most prevalent mutations associated with resistance to antiretroviral drugs (ARV), where the major pattern included regional dispersal with some cases of cross-regional transmissions [21,53]. Similar findings were reported elsewhere, highlighting the important effect of transmission clusters in the increasing prevalence of NNRTI resistance mutations among treatment-naïve populations [37,51,54,55,56].

In conclusion, our study provides a detailed picture of the characteristics of the HIV-1 epidemic regarding the dispersal patterns of the most prevalent subtypes in Greece. Since we are one step before the implementation of molecular surveillance as a method to identify PLHIV and their links to higher risk for HIV infection, our aim was to perform a comprehensive analysis of MTCs and their characteristics. We identified the MTCs using a novel approach for bootstrapping and we showed that HIV-1 regional transmission dominates in Greece and, also, it is associated with the MSM risk group (subtype A1) and Greek origin (both subtypes). Overall, our findings provide some novel insights that can be important for national policy against HIV. Moreover, we showed that approximately one-quarter of introductions leads to regional dispersal. This estimate might provide a proxy of the percentage of PLHIV contributed to onward transmissions.

## Figures and Tables

**Table 1:** Demographic characteristics of a subset of the study population (i.e., PLHIV infected with subtypes A1 and B).

Characteristic	Subtype A1	Subtype B
Age, years, median (IQR)	37 (30–46) ^1^	36 (30–44) ^2^
Gender, *n* (%)		
Male	1.435 (81.9)	2217 (86.1)
Female	232 (13.3)	282 (10.9)
Unknown	84 (4.8)	76 (3.0)
Transmission risk group, *n* (%)		
MSM	1035 (59.1)	1718 (66.7)
PWID	150 (8.6)	182 (7.1)
Heterosexuals	387 (22.1)	438 (17.0)
Other	20 (1.1)	88 (3.4)
Unknown	159 (9.1)	149 (5.8)
Origin, *n* (%)		
Greek	1321 (75.4)	2117 (82.2)
Non-Greek	197 (11.3)	186 (7.2)
Unknown	233 (13.3)	272 (10.6)
Sampling period		
1999–2005	365 (20.8)	1020 (39.6)
2006–2010	503 (28.7)	618 (24.0)
2011–2015	880 (50.3)	934 (36.3)
Unknown	3 (0.2)	3 (0.1)
Total, *n*, (%)	1751 (100.0)	2575 (100.0)

^1^*n* = 1642 (of 1751 PLHIV); ^2^ *n* = 2457 (of 2575 PLHIV). PLHIV—people living with HIV; IQR—interquartile range; MSM—men having sex with men; PWID—people who inject drugs.

**Table 2:** Demographic characteristics of PLHIV who were infected with subtypes A1 and B within and outside molecular transmission clusters (MTCs).

	Subtype A1		Subtype B	
Characteristic	Within MTCs	Outside MTCs	Within MTCs	Outside MTCs
Age, years, median (IQR)	37 (29–46) ^1^	37 (31–48) ^2^	35 (29–42) ^3^	40 (34–48) ^4^
Gender, *n* (%)				
Male	1373 (83.6)	62 (56.9)	1729 (87.2)	488 (82.3)
Female	193 (11.8)	39 (35.8)	196 (9.9)	86 (14.5)
Unknown	76 (4.6)	8 (7.3)	57 (2.9)	19 (3.2)
Transmission risk group, *n* (%)				
MSM	1022 (62.2)	13 (11.9)	1373 (69.3)	345 (58.2)
PWID	124 (7.6)	26 (23.9)	148 (7.5)	34 (5.7)
Heterosexuals	339 (20.6)	48 (44.0)	321 (16.2)	117 (19.7)
Other	18 (1.1)	2 (1.8)	23 (1.1)	65 (11.0)
Unknown	139 (8.5)	20 (18.4)	117 (5.9)	32 (5.4)
Origin, *n* (%)				
Greek	1283 (78.1)	38 (34.9)	1635 (82.5)	482 (81.3)
Non-Greek	150 (9.2)	47 (43.1)	139 (7.0)	47 (7.9)
Unknown	209 (12.7)	24 (22.0)	208 (10.5)	64 (10.8)
Sampling period				
[1999–2005]	350 (21.3)	15 (13.7)	647 (32.6)	373 (62.9)
[2006–2010]	459 (28.0)	44 (40.4)	510 (25.7)	108 (18.2)
[2011–2015]	830 (50.5)	50 (45.9)	822 (41.5)	112 (18.9)
Unknown	3 (0.2)	0 (0.0)	3 (0.2)	0 (0.0)
Total, *n*, (%)	1642 (100.0)	109 (100.0)	1982 (100.0)	593 (100.0)

^1^*n* = 1540 (of 1642 PLHIV); ^2^ *n* = 102 (of 109 PLHIV); ^3^ *n* = 1891 (of 1982 PLHIV); ^4^ *n* = 566 (of 593 PLHIV). PLHIV—people living with HIV; IQR—interquartile range; MSM—men having sex with men; PWID—people who inject drugs.

**Table 3:** Multivariable logistic regression estimates using the presence in molecular transmission clusters (MTCs) as the binary outcome variable.

	Subtype A1 ^1^			Subtype B ^1^		
Explanatory Variable	Odds Ratio	95% CI	*p*-Value	Odds Ratio	95% CI	*p*-Value
Age	0.99	0.97–1.01	0.341	0.96	0.95–0.97	<0.001
Gender						
Male	1.52	0.87–2.67	0.140	1.33	0.91–1.97	0.141
Female ^2^						
Unknown	2.80	0.64–12.3	0.172	0.84	0.22–3.16	0.796
Transmission risk group						
MSM	5.34	2.52–11.31	<0.001	0.98	0.69–1.38	0.893
PWID	0.42	0.22–0.80	0.008	0.80	0.49–1.30	0.364
Heterosexuals ^2^						
Other	0.86	0.17–4.47	0.861	0.10	0.05–0.18	<0.001
Unknown	0.81	0.37–1.80	0.610	0.96	0.48–1.95	0.919
Origin						
Greek	6.05	3.62–10.10	<0.001	1.57	1.08–2.29	0.019
Non-Greek ^2^						
Unknown	1.27	0.62–2.60	0.516	1.26	0.74–2.12	0.394
Sampling period						
[1999–2005] ^2^						
[2006–2010]	0.51	0.25–1.01	0.054	2.33	1.80–3.02	<0.001
[2011–2015]	0.75	0.38–1.48	0.411	3.83	2.95–4.96	<0.001
Unknown	1	-	-	1	-	-
Sampling area						
Central/Southern Greece ^2^						
Northern Greece	0.91	0.54–1.55	0.736	1.32	0.96–1.81	0.092
Crete	1	-	-	1	-	-

^1^ Each model was fit to a subset of the original data (A1: 1751; B: 2575) consisting of 1635 subtype A1 and 2453 subtype B complete observations; ^2^ Reference category. CI—confidence interval; MSM—men having sex with men; PWID—people who inject drugs.

**Table 4:** Migration matrix representing the mean number of viral migrations events (transmissions) among the different geographic areas in Greece across all the phylogenetic bootstrap trees (*n* = 408), separately for each HIV-1 subtype under study.

	Subtype A1		
	Importing to		
Exporting from	Central/Southern Greece	Northern Greece	Crete
Central/Southern Greece		90.9	11.6
Northern Greece	**35.4** ^1^		0.2
Crete	**0.1** ^1^	0	
	**Subtype B**		
Central/Southern Greece		67.6	15.0
Northern Greece	**17.4** ^1^		**1.4** ^1^
Crete	**1.2** ^1^	**0.5** ^1^	

^1^ Statistically significant pathways (compared with the null hypothesis representing the simulated scenario of panmixis). The level of significance has been adjusted according to the Bonferroni correction for multiple comparisons.

## Supplementary Materials

The following is available online at https://www.mdpi.com/1999-4915/12/10/1183/s1, Figure S1: Distribution of number of sequences per molecular transmission cluster (MTC) for: (a) subtype A1 and (b) subtype B.

## References

[1] Foley B.T., Leitner T., Paraskevis D., Peeters M. (2016). Primate immunodeficiency virus classification and nomenclature: Review. Infect. Genet. Evol..

[2] Yamaguchi J., Vallari A., McArthur C., Sthreshley L., Cloherty G.A., Berg M.G., Rodgers M.A. (2020). Complete Genome Sequence of CG-0018a-01 Establishes HIV-1 Subtype L. J. Acquir Immune Defic. Syndr..

[3] Hemelaar J., Elangovan R., Yun J., Dickson-Tetteh L., Fleminger I., Kirtley S., Williams B., Gouws-Williams E., Ghys P.D., Abimiku A.l.G. (2019). Global and regional molecular epidemiology of HIV-1, 1990–2015: A systematic review, global survey, and trend analysis. Lancet Infect. Dis..

[4] Abeler-Dorner L., Grabowski M.K., Rambaut A., Pillay D., Fraser C., PANGEA Consortium (2019). PANGEA-HIV 2: Phylogenetics And Networks for Generalised Epidemics in Africa. Curr. Opin. HIV AIDS.

[5] Van de Vijver D., Boucher C.A.B. (2018). Insights on transmission of HIV from phylogenetic analysis to locally optimize HIV prevention strategies. Curr. Opin. HIV AIDS.

[6] Brenner B.G., Ibanescu R.I., Hardy I., Roger M. (2017). Genotypic and Phylogenetic Insights on Prevention of the Spread of HIV-1 and Drug Resistance in “Real-World” Settings. Viruses.

[7] Chaillon A., Essat A., Frange P., Smith D.M., Delaugerre C., Barin F., Ghosn J., Pialoux G., Robineau O., Rouzioux C. (2017). Spatiotemporal dynamics of HIV-1 transmission in France (1999–2014) and impact of targeted prevention strategies. Retrovirology.

[8] Grabowski M.K., Lessler J. (2017). Phylogenetic insights into age-disparate partnerships and HIV. Lancet HIV.

[9] Bezemer D., Cori A., Ratmann O., van Sighem A., Hermanides H.S., Dutilh B.E., Gras L., Rodrigues Faria N., van den Hengel R., Duits A.J. (2015). Dispersion of the HIV-1 Epidemic in Men Who Have Sex with Men in the Netherlands: A Combined Mathematical Model and Phylogenetic Analysis. PLoS Med..

[10] Vasylyeva T.I., Zarebski A., Smyrnov P., Williams L.D., Korobchuk A., Liulchuk M., Zadorozhna V., Nikolopoulos G., Paraskevis D., Schneider J. (2020). Phylodynamics Helps to Evaluate the Impact of an HIV Prevention Intervention. Viruses.

[11] Paraskevis D., Nikolopoulos G.K., Magiorkinis G., Hodges-Mameletzis I., Hatzakis A. (2016). The application of HIV molecular epidemiology to public health. Infect. Genet. Evol..

[12] Brenner B.G., Wainberg M.A. (2013). Future of phylogeny in HIV prevention. J. Acquir. Immune Defic. Syndr..

[13] Sivay M.V., Grabowski M.K., Zhang Y., Palumbo P.J., Guo X., Piwowar-Manning E., Hamilton E.L., Viet Ha T., Antonyak S., Imran D. (2019). Phylogenetic Analysis of Human Immunodeficiency Virus from People Who Inject Drugs in Indonesia, Ukraine, and Vietnam: HPTN 074. Clin. Infect. Dis..

[14] Alpren C., Dawson E.L., John B., Cranston K., Panneer N., Fukuda H.D., Roosevelt K., Klevens R.M., Bryant J., Peters P.J. (2020). Opioid Use Fueling HIV Transmission in an Urban Setting: An Outbreak of HIV Infection Among People Who Inject Drugs-Massachusetts, 2015–2018. Am. J. Public Health.

[15] Kostaki E.G., Frampton D., Paraskevis D., Pantavou K., Ferns B., Raffle J., Grant P., Kozlakidis Z., Hadjikou A., Pavlitina E. (2018). Near Full-length Genomic Sequencing and Molecular Analysis of HIV-Infected Individuals in a Network-based Intervention (TRIP) in Athens, Greece: Evidence that Transmissions Occur More Frequently from those with High HIV-RNA. Curr. HIV Res..

[16] Peters P.J., Pontones P., Hoover K.W., Patel M.R., Galang R.R., Shields J., Blosser S.J., Spiller M.W., Combs B., Switzer W.M. (2016). HIV Infection Linked to Injection Use of Oxymorphone in Indiana, 2014–2015. N. Engl. J. Med..

[17] CDC: Detecting and Responding to HIV Transmission Clusters. A Guide for Health Departments. https://www.cdc.gov/hiv/pdf/funding/announcements/ps18-1802/CDC-HIV-PS18-1802-AttachmentE-Detecting-Investigating-and-Responding-to-HIV-Transmission-Clusters.pdf.

[18] Paraskevis D., Beloukas A., Stasinos K., Pantazis N., de Mendoza C., Bannert N., Meyer L., Zangerle R., Gill J., Prins M. (2019). HIV-1 molecular transmission clusters in nine European countries and Canada: Association with demographic and clinical factors. BMC Med..

[19] Paraskevis D., Magiorkinis E., Magiorkinis G., Sypsa V., Paparizos V., Lazanas M., Gargalianos P., Antoniadou A., Panos G., Chrysos G. (2007). Increasing prevalence of HIV-1 subtype A in Greece: Estimating epidemic history and origin. J. Infect. Dis..

[20] Paraskevis D., Paraschiv S., Sypsa V., Nikolopoulos G., Tsiara C., Magiorkinis G., Psichogiou M., Flampouris A., Mardarescu M., Niculescu I. (2015). Enhanced HIV-1 surveillance using molecular epidemiology to study and monitor HIV-1 outbreaks among intravenous drug users (IDUs) in Athens and Bucharest. Infect. Genet. Evol..

[21] Paraskevis D., Kostaki E., Magiorkinis G., Gargalianos P., Xylomenos G., Magiorkinis E., Lazanas M., Chini M., Nikolopoulos G., Skoutelis A. (2017). Prevalence of drug resistance among HIV-1 treatment-naive patients in Greece during 2003–2015: Transmitted drug resistance is due to onward transmissions. Infect. Genet. Evol..

[22] NPHO: HIV/AIDS Surveillance in Greece Diagnoses through 31/12/2019. https://eody.gov.gr/wp-content/uploads/2018/12/epidimiologiko-deltio-hiv-2019.pdf.

[23] Kumar S., Stecher G., Li M., Knyaz C., Tamura K. (2018). MEGA X: Molecular Evolutionary Genetics Analysis across Computing Platforms. Mol. Biol. Evol..

[24] Price M.N., Dehal P.S., Arkin A.P. (2010). FastTree 2—Approximately maximum-likelihood trees for large alignments. PLoS ONE.

[25] Lole K.S., Bollinger R.C., Paranjape R.S., Gadkari D., Kulkarni S.S., Novak N.G., Ingersoll R., Sheppard H.W., Ray S.C. (1999). Full-Length Human Immunodeficiency Virus Type 1 Genomes from Subtype C-Infected Seroconverters in India, with Evidence of Intersubtype Recombination. J. Virol..

[26] Stamatakis A. (2014). RAxML version 8: A tool for phylogenetic analysis and post-analysis of large phylogenies. Bioinformatics.

[27] Kostaki E.G., Flampouris A., Karamitros T., Chueca N., Alvarez M., Casas P., Alejos B., Hatzakis A., Garcia F., Paraskevis D. (2019). Spatiotemporal Characteristics of the Largest HIV-1 CRF02_AG Outbreak in Spain: Evidence for Onward Transmissions. Front. Microbiol..

[28] Von Wyl V., Kouyos R.D., Yerly S., Boni J., Shah C., Burgisser P., Klimkait T., Weber R., Hirschel B., Cavassini M. (2011). The role of migration and domestic transmission in the spread of HIV-1 non-B subtypes in Switzerland. J. Infect. Dis..

[29] Lemoine F., Domelevo Entfellner J.B., Wilkinson E., Correia D., Davila Felipe M., De Oliveira T., Gascuel O. (2018). Renewing Felsenstein’s phylogenetic bootstrap in the era of big data. Nature.

[30] Ronquist F., Teslenko M., van der Mark P., Ayres D.L., Darling A., Hohna S., Larget B., Liu L., Suchard M.A., Huelsenbeck J.P. (2012). MrBayes 3.2: Efficient Bayesian phylogenetic inference and model choice across a large model space. Syst. Biol..

[31] Rambaut A., Drummond A.J., Xie D., Baele G., Suchard M.A. (2018). Posterior Summarization in Bayesian Phylogenetics Using Tracer 1.7. Syst. Biol..

[32] Maddison W.P., Maddison D.R. Mesquite: A Modular System for Evolutionary Analysis. Version 3.61. http://www.mesquiteproject.org.

[33] Wilgenbusch J.C., Swofford D. (2003). Inferring evolutionary trees with PAUP*. Curr. Protoc. Bioinform..

[34] Angelis K., Albert J., Mamais I., Magiorkinis G., Hatzakis A., Hamouda O., Struck D., Vercauteren J., Wensing A.M., Alexiev I. (2015). Global Dispersal Pattern of HIV Type 1 Subtype CRF01_AE: A Genetic Trace of Human Mobility Related to Heterosexual Sexual Activities Centralized in Southeast Asia. J. Infect. Dis..

[35] Magiorkinis G., Angelis K., Mamais I., Katzourakis A., Hatzakis A., Albert J., Lawyer G., Hamouda O., Struck D., Vercauteren J. (2016). The global spread of HIV-1 subtype B epidemic. Infect. Genet. Evol..

[36] Paraskevis D., Kostaki E., Nikolopoulos G.K., Sypsa V., Psichogiou M., Del Amo J., Hodges-Mameletzis I., Paraskeva D., Skoutelis A., Malliori M. (2017). Molecular Tracing of the Geographical Origin of Human Immunodeficiency Virus Type 1 Infection and Patterns of Epidemic Spread Among Migrants Who Inject Drugs in Athens. Clin. Infect. Dis..

[37] Fabeni L., Santoro M.M., Lorenzini P., Rusconi S., Gianotti N., Costantini A., Sarmati L., Antinori A., Ceccherini-Silberstein F., d’Arminio Monforte A. (2020). Evaluation of HIV Transmission Clusters among Natives and Foreigners Living in Italy. Viruses.

[38] Verhofstede C., Mortier V., Dauwe K., Callens S., Deblonde J., Dessilly G., Delforge M.L., Fransen K., Sasse A., Stoffels K. (2019). Exploring HIV-1 Transmission Dynamics by Combining Phylogenetic Analysis and Infection Timing. Viruses.

[39] Kostrikis L.G., Hezka J., Stylianou D.C., Kostaki E., Andreou M., Kousiappa I., Paraskevis D., Demetriades I. (2018). HIV-1 transmission networks across Cyprus (2010–2012). PLoS ONE.

[40] Lewis F., Hughes G.J., Rambaut A., Pozniak A., Brown A.J.L. (2008). Episodic Sexual Transmission of HIV Revealed by Molecular Phylodynamics. PLoS Med..

[41] Beloukas A., Psarris A., Giannelou P., Kostaki E., Hatzakis A., Paraskevis D. (2016). Molecular epidemiology of HIV-1 infection in Europe: An overview. Infect. Genet. Evol..

[42] Ciccozzi M., Gori C., Boros S., Ruiz-Alvarez M.J., Harxhi A., Dervishi M., Qyra S., Schinaia N., D’Arrigo R., Ceccherini-Silberstein F. (2005). Molecular Diversity of HIV in Albania. J. Infect. Dis..

[43] Aibekova L., Foley B., Hortelano G., Raees M., Abdraimov S., Toichuev R., Ali S. (2018). Molecular epidemiology of HIV-1 subtype A in former Soviet Union countries. PLoS ONE.

[44] Schlosser M., Kartashev V.V., Mikkola V.H., Shemshura A., Saukhat S., Kolpakov D., Suladze A., Tverdokhlebova T., Hutt K., Heger E. (2020). HIV-1 Sub-Subtype A6: Settings for Normalised Identification and Molecular Epidemiology in the Southern Federal District, Russia. Viruses.

[45] Bobkov A., Cheingsong-Popov R., Selimova L., Ladnaya N., Kazennova E., Kravchenko A., Fedotov E., Saukhat S., Zverev S., Pokrovsky V. (1997). An HIV Type 1 Epidemic among Injecting Drug Users in the Former Soviet Union Caused by a Homogeneous Subtype A Strain. AIDS Res. Hum. Retrovir..

[46] Thomson M.M., de Parga E.V., Vinogradova A., Sierra M., Yakovlev A., Rakhmanova A., Delgado E., Casado G., Munoz M., Carmona R. (2007). New insights into the origin of the HIV type 1 subtype A epidemic in former Soviet Union’s countries derived from sequence analyses of preepidemically transmitted viruses. AIDS Res. Hum. Retrovir..

[47] Diez-Fuertes F., Cabello M., Thomson M.M. (2015). Bayesian phylogeographic analyses clarify the origin of the HIV-1 subtype A variant circulating in former Soviet Union’s countries. Infect. Genet. Evol..

[48] Araujo P.M.M., Carvalho A., Pingarilho M., Abecasis A.B., Osorio N.S. (2019). Characterization of a large cluster of HIV-1 A1 infections detected in Portugal and connected to several Western European countries. Sci. Rep..

[49] Abecasis A.B., Wensing A.M.J., Paraskevis D., Vercauteren J., Theys K., Van de Vijver D.A.M.C., Albert J., Asjö B., Balotta C., Beshkov D. (2013). HIV-1 subtype distribution and its demographic determinants in newly diagnosed patients in Europe suggest highly compartmentalized epidemics. Retrovirology.

[50] Aldous J.L., Pond S.K., Poon A., Jain S., Qin H., Kahn J.S., Kitahata M., Rodriguez B., Dennis A.M., Boswell S.L. (2012). Characterizing HIV transmission networks across the United States. Clin. Infect. Dis..

[51] Drescher S.M., von Wyl V., Yang W.L., Boni J., Yerly S., Shah C., Aubert V., Klimkait T., Taffe P., Furrer H. (2014). Treatment-naive individuals are the major source of transmitted HIV-1 drug resistance in men who have sex with men in the Swiss HIV Cohort Study. Clin. Infect. Dis..

[52] Kostaki E.G., Soulie C., Visseaux B., Storto A., Charpentier C., Wirden M., Landman R., Katlama C., Calvez V., Descamps D. Molecular Analysis Suggests Post-Migration HIV-1 Acquisition among Migrants in Paris. Proceedings of the Virtual Conference on Retroviruses and Opportunistic Infections.

[53] Skoura L., Metallidis S., Buckton A.J., Mbisa J.L., Pilalas D., Papadimitriou E., Papoutsi A., Haidich A.B., Chrysanthidis T., Tsachouridou O. (2011). Molecular and epidemiological characterization of HIV-1 infection networks involving transmitted drug resistance mutations in Northern Greece. J. Antimicrob. Chemother..

[54] Fabeni L., Alteri C., Di Carlo D., Orchi N., Carioti L., Bertoli A., Gori C., Forbici F., Continenza F., Maffongelli G. (2017). Dynamics and phylogenetic relationships of HIV-1 transmitted drug resistance according to subtype in Italy over the years 2000-14. J. Antimicrob. Chemother..

[55] Rhee S.Y., Blanco J.L., Jordan M.R., Taylor J., Lemey P., Varghese V., Hamers R.L., Bertagnolio S., Rinke de Wit T.F., Aghokeng A.F. (2015). Geographic and temporal trends in the molecular epidemiology and genetic mechanisms of transmitted HIV-1 drug resistance: An individual-patient- and sequence-level meta-analysis. PLoS Med..

[56] Kleyn T.J., Liedtke M.D., Harrison D.L., Lockhart S.M., Salvaggio M.R., Ripley T.L., Rathbun R.C. (2014). Incidence of transmitted antiretroviral drug resistance in treatment-naive HIV-1-infected persons in a large South Central United States clinic. Ann. Pharm..

