# Supplementary figures and images for: A Nationwide Study about the Dispersal Patterns of the Predominant HIV-1 Subtypes A1 and B in Greece: Inference of the Molecular Transmission Clusters
Source: Viruses. 2020 Oct 19;12(10):1183. doi: 10.3390/v12101183 (PMC7589601; doi:10.3390/v12101183)

(a)

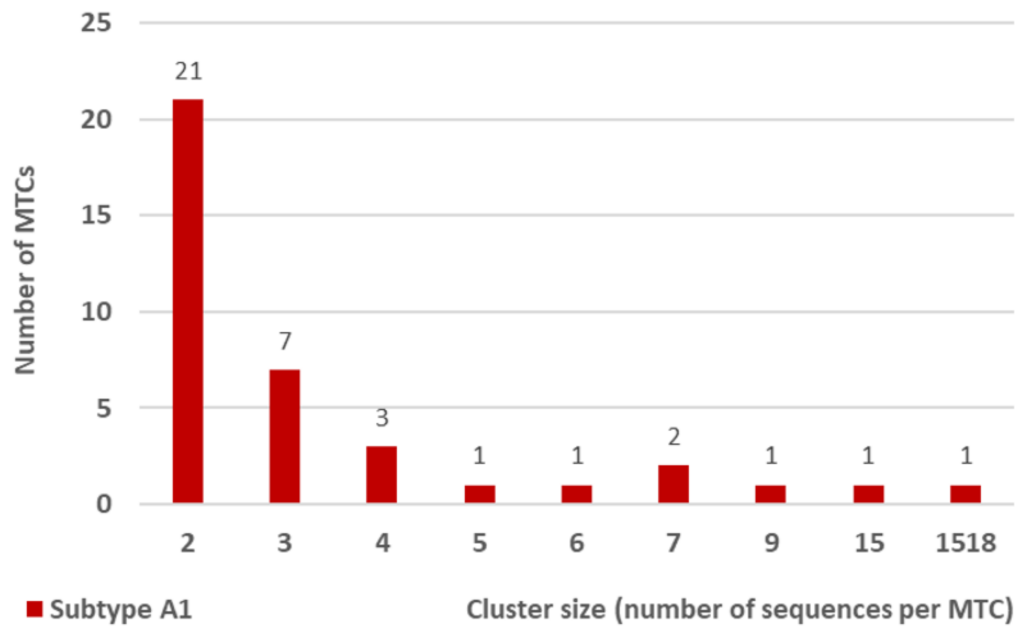

(b)

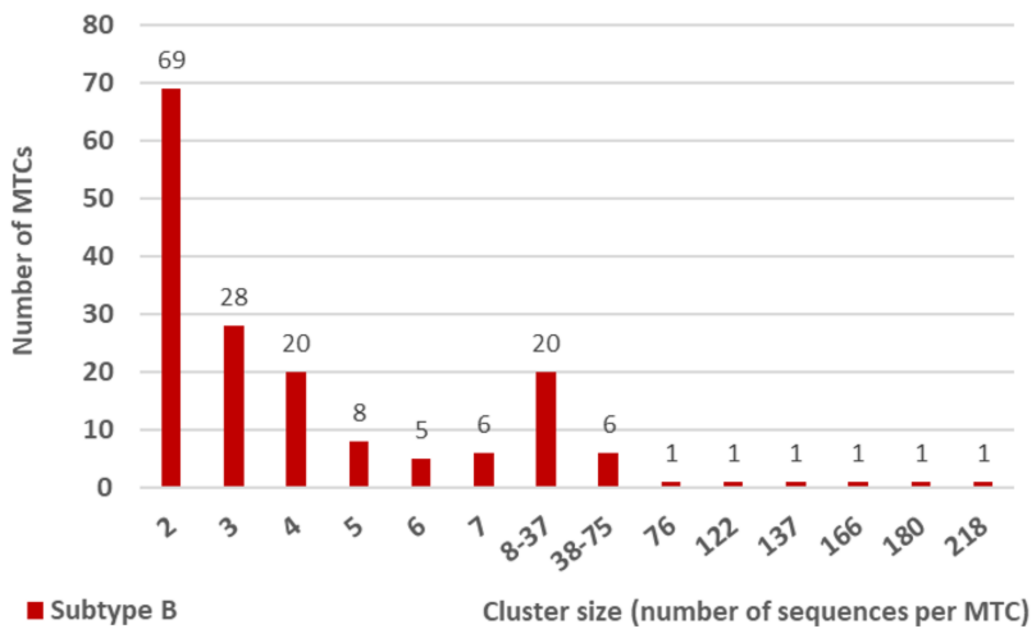

Supplement: Supplementary file 1 [file viruses-12-01183-s001.pdf]
